# Supplementary material for: MetaQTL: a package of new computational methods for the meta-analysis of QTL mapping experiments
Source: BMC Bioinformatics. 2007 Feb 8;8:49. doi: 10.1186/1471-2105-8-49 (PMC1808479; doi:10.1186/1471-2105-8-49)
Supplement: Additional File 7 — MetaQTL Package : jar file and tutorial. This Zip archive contains both the MetaQTL JAR file and the files of the tutorial. [file 1471-2105-8-49-S7.zip › org.inra.metaqtl/doc/org/metaqtl/algo/QtlClustAlgorithm.html]

QtlClustAlgorithm


|  |  |  |
| --- | --- | --- |
| **Overview** \| **Package** \| **Class** \| **Use** \| **Tree** \| **Deprecated** \| **Index** \| **Help** | |  |
| **PREV CLASS**   **NEXT CLASS** | **FRAMES**    **NO FRAMES**     **All Classes** |  |
| SUMMARY: NESTED \| FIELD \| CONSTR \| METHOD | DETAIL: FIELD \| CONSTR \| METHOD |  |


---


## org.metaqtl.algo Class QtlClustAlgorithm

```
java.lang.Object
  org.metaqtl.algo.MetaAlgorithm
      org.metaqtl.algo.ClustAlgorithm
          org.metaqtl.algo.QtlClustAlgorithm
```

**All Implemented Interfaces:**: IMetaAlgorithm

---

``` public class QtlClustAlgorithm extends ClustAlgorithm ```

This class defines methods to perform a QTL meta-analysis
on a genetic map by applying a clustering of QTL
based on a gaussian mixture model and the EM-algorihtm.

---

**Field Summary**

**Fields inherited from class org.metaqtl.algo.ClustAlgorithm**

`chrName, chromosomes, missing_sd_mode, ontology, result, sd_mode`

**Fields inherited from class org.metaqtl.algo.MetaAlgorithm**

`logger, loggerUp, workAmount, workProgress`


**Constructor Summary**

`QtlClustAlgorithm(IBioGenome map,
IBioOntology ontology)`

`QtlClustAlgorithm(IBioGenome map,
java.lang.String chrom,
IBioOntology ontology)`


| **Method Summary** | |
| --- | --- |
| `MetaQtlResult[]` | `doQtlClustering(MetaQtlData data)`             For a given data set `data` performs the clustering on each trait group and returns the results as an array of `MetaQtlResult` objects. |
| `double` | `getEMEps()` |
| `int` | `getEMRanStartNumber()` |
| `void` | `run()`             This method performs a QTL meta-analysis on each chromosome of the given map. |
| `void` | `setEMEps(double epsilon)` |
| `void` | `setEMRanStartNumber(int n)` |
| `void` | `setMaxClusterNumber(int nmax)` |

**Methods inherited from class org.metaqtl.algo.ClustAlgorithm**

`getResult, setCIMiss, setCIMode`

**Methods inherited from class org.metaqtl.algo.MetaAlgorithm**

`disableLogger, getLogger, getWorkAmount, getWorkProgress, isLoggerEnable, setLogger, setLogger`

**Methods inherited from class java.lang.Object**

`clone, equals, finalize, getClass, hashCode, notify, notifyAll, toString, wait, wait, wait`

**Constructor Detail**

### QtlClustAlgorithm

```
public QtlClustAlgorithm(IBioGenome map,
                         IBioOntology ontology)
```

**Parameters:**: `map` -: `ontology` -

---


### QtlClustAlgorithm

```
public QtlClustAlgorithm(IBioGenome map,
                         java.lang.String chrom,
                         IBioOntology ontology)
```


**Method Detail**

### run

```
public void run()
```

:   This method performs a QTL meta-analysis on each chromosome
    of the given map. For each chromosome all the possible
    QTL cluster combinations are tested and the results are
    added to the chromosome. Then the method returns the array
    of chromosomes for which the meta-analysis has been performed.

---


### doQtlClustering

```
public MetaQtlResult[] doQtlClustering(MetaQtlData data)
```

:   For a given data set `data` performs the clustering
    on each trait group and returns the results as an array of
    `MetaQtlResult` objects.

    :   **Parameters:**: `data` - the data set. **Returns:**: the results of teh clustering. **See Also:**: `MetaQtlData`, `MetaQtlResult`

---


### setMaxClusterNumber

```
public void setMaxClusterNumber(int nmax)
```

:   **Parameters:**: `nmax` - the maximum number of clusters to test.

---


### getEMRanStartNumber

```
public int getEMRanStartNumber()
```

:   **Returns:**: Returns the number of random starting points.

---


### setEMRanStartNumber

```
public void setEMRanStartNumber(int n)
```

:   **Parameters:**: `n_em_start` - The n\_em\_start to set.

---


### getEMEps

```
public double getEMEps()
```

:   **Returns:**: Returns the convergence error for the EM.

---


### setEMEps

```
public void setEMEps(double epsilon)
```

:   **Parameters:**: `em_err` - The em\_err to set.


---


|  |  |  |
| --- | --- | --- |
| **Overview** \| **Package** \| **Class** \| **Use** \| **Tree** \| **Deprecated** \| **Index** \| **Help** | |  |
| **PREV CLASS**   **NEXT CLASS** | **FRAMES**    **NO FRAMES**     **All Classes** |  |
| SUMMARY: NESTED \| FIELD \| CONSTR \| METHOD | DETAIL: FIELD \| CONSTR \| METHOD |  |


---
